# Socioeconomic inequality in unmet outpatient healthcare needs among people living in urban informal settlements in Sanandaj city, Iran

**DOI:** 10.1186/s12939-023-02076-1

**Published:** 2023-12-11

**Authors:** Bakhtiar Piroozi, Hossein Safari, Amjad Mohamadi Bolbanabad, Ghobad Moradi, Yadolah Zarezadeh, Azad Shokri, Farhad Moradpour

**Affiliations:** 1 Social Determinants of Health Research Center, Research Institute for Health Development, Kurdistan University of Medical Sciences, Sanandaj, Iran; 2 Health Promotion Research Center, Iran University of Medical Sciences, Tehran, Iran

**Keywords:** Unmet healthcare needs, Access, Informal settlements, Health inequality

## Abstract

**Background:**

The growing trend of informal settlements is a serious humanitarian crisis. Unmet need for health care services is an indicator to measure the state of equality and access to healthcare services. This study, for the first time in Iran, examined the prevalence of unmet needs for outpatient healthcare services and related socioeconomic inequalities among residents of informal settlements in Sanandaj city.

**Methods:**

This cross-sectional study was conducted on informal settlements of Sanandaj city with a sample size of 1345 people. Data were collected using a questionnaire. Multivariate logistic regression was used to determine significant predictors of unmet needs for healthcare services. Concentration index (C) and concentration curve (CC) were calculated to measure inequality in the prevalence of unmet needs for healthcare services.

**Results:**

The prevalence of unmet needs for outpatient healthcare services was 31.7%. Financial and physical barriers were the most common reasons for not using the needed services. The highest unmet need was related to dental (80.6%) and rehabilitation services (78.8%). Being elderly with about 2.3 times (OR: 2.37, 95% CI: 1.19–4.75), not having a job with about 1.7 times (OR: 1.70, 95% CI: 1.13–2.57) and having a low economic status with about 4 times (OR: 4.46, 95% CI: 2.39–9.70) increased the odds of experiencing unmet need for outpatient healthcare services. The value of concentration index showed that unmet need for outpatient healthcare services was significantly concentrated among people with lower economic status (C= -0.330, 95% CI: -0.432 to -0.227).

**Conclusion:**

The unmet need is high among people living in informal settlements of Sanandaj city and a significant part of the residents of these settlements does not have access to required healthcare services. Regardless of the needs of people living in these settlements, who constitute a large population of Iran, access to universal health coverage is not possible in such areas. Removing the identified obstacles and causes behind the unmet needs requires the interdisciplinary participation of all actors, including the government, the nation, and civil society.

## Introduction

The global rise of urbanization is linked to past challenges. Prioritizing economic, social, and environmental resilience with effective governance is crucial for having sustainable cities. Urbanization will continue with the urban population projected to reach 68% by 2050 [1]. Informal settlements and marginalization status are one of the social problems of human societies [2]. Informal settlements are places created without permission and outside the official and legal planning of urban development that do not comply with authorized regulations [3]. As reported by the United Nations, more than one billion of the world’s population and about 43% of the population of urban areas in developing countries live in informal settlements. The massive growth of informal settlements has become a major challenge and debate in sustainable development of cities. Paying attention to sustainable development of cities is very important in reaching Sustainable Development Goals (SDG) of the United Nations. Goal number 11 focuses on transforming cities and informal settlements into inclusive, safe, resilient and sustainable places, which is itself related to goal number 3 that deals with providing a healthy life and promoting well-being for all ages [4].

Informal settlements do not have a suitable condition in terms of social determinants of health, and this can result in social damages and diseases [5]. People living in informal settlements are exposed to economic and social inequalities in accessing and utilizing healthcare services and experiencing health outcomes [6]. Residents of informal settlements face challenges such as lack of proper access to healthcare services; shortage of centers providing healthcare services; extreme poverty; unemployment; illiteracy and low education; gender discrimination; insufficient and poor diet; social exclusion; unhealthy living environment; non-standard housing; lack of water, electricity and basic sanitary facilities; lack of educational and recreational facilities; and high prevalence of diseases [7–9].

In Iran, urbanization without proper planning causes the creation of informal settlements around the cities over time [10]. According to statistics, about 11 million people of Iran live in informal settlements [11]. Marginalization can be seen in many regions of Iran. Kurdistan province is one of the provinces with the highest ratio of marginalization in Iran, so that the ratio of marginalized population in this province is three times the national average and more than half of the urban population in this province lives in marginal and isolated urban areas [12].

Providing evidence of status of need, access and unmet healthcare needs along with their causes in informal settlements is very important in order to design interventions, have planning and public health policy [13, 14]. In developing countries, information related to situation and needs of marginalized and vulnerable groups is less documented. Unmet healthcare needs are the difference between the health care services people need and the services people actually receive [15]. Unmet healthcare needs play an important role in creating health inequalities [16].

Identifying the gap between “perceived needs for healthcare services” and “use of healthcare services” and recognizing the causes of this gap is very important in order to design targeted interventions to reduce it and improve access to health care services [15].

To the best of our knowledge, no study has ever been done on investigating unmet healthcare needs in informal settlements in Iran. Also, there is not adequate evidence for evidence-based policy-making in this regard. Thus, our research questions included: What is the prevalence of unmet outpatient healthcare needs in informal settlements in Sanandaj city (the capital of Kurdistan province)? What are the reasons behind these unmet outpatient healthcare needs? What are the socio-demographic and contextual factors related to these unmet outpatient healthcare needs?

## Methods and materials

This descriptive-analytical and cross-sectional study was conducted on households living in informal settlements of Sanandaj city with a population of about 500 thousand people as the capital of Kurdistan province in west of Iran. The study population included 1354 people by taking into account p = 17% (unmet outpatient healthcare needs) [20], d = 0.02 (accuracy level) and α = 0.05 (type 1 error). We collected files and data of the households in health centers of informal settlements of Sanandaj city (including the areas of Nayser, Nanleh, Hasan Abad, Asaule, Dushan and Ghareza). In this study, we used stratified random sampling, where each center was proportionally represented based on its size in total population. Households were selected in two phases: first, each household was assigned a unique identification number, and then they were selected based on random number generator in Excel software. After going to the door of the house, the questionnaire of benefiting from health services was completed for the first person over 18 years of age who was available.

This standard questionnaire has been used in several national and provincial studies in Iran [15]. Finally, 1346 people (response rate: 99%) were interviewed. Participants were asked about the need for outpatient healthcare services in the last 30 days. Next, people with a history of need were asked questions about using these services. History of need for outpatient health care services was obtained from the following question: During the past 30 days, was there a time when you needed outpatient healthcare services? Respondents who required outpatient health care services (n = 505) were included in this study. People who did not use health care services were asked about the reasons for not using such services.

### Variables

In this study, unmet outpatient healthcare needs as an outcome variable (have or don’t have) was classified based on demographic and background variables including gender (male or female), age (under 30 years, 30 to 59 or over 60 years), education (illiterate, below diploma, diploma or university degree), employment status (employed or unemployed), basic health insurance status (have or don’t have), supplementary health insurance status (have or don’t have), and economic status. The economic status was defined using a wealth index. This Index was calculated based on selected household assets including ownership of a computer/laptop, dishwasher, washing machine, air conditioner, vacuum cleaner, microwave, color TV, types of regular travel, car and home ownership. Principal component analysis (PCA) was conducted on asset ownership data to generate total wealth scores for participants. Participants were then ranked according to their wealth scores. Natural breakpoints in the distribution were identified using quantiles, which divided the range into five probability groups with equal proportions. The resulting three categories formed the wealth index groups: “poor”, “middle”, and “rich”. This allowed the classification of participants’ economic status based on their household assets and possessions.

### Statistical analysis

As widely used in previous studies [17, 18], data including having computer/laptop, dishwasher, washing machine, air conditioner, vacuum cleaner, microwave, color TV, travelling, owning a car and house were used to create an economic status by the help of principal component analysis (PCA). We also used Pearson’s chi-square test in order to clarify the differences between the respondents regarding unmet and met outpatient healthcare needs. In addition, the determinants of unmet outpatient healthcare needs were analyzed using a logistic regression analysis with maximum likelihood. The odds ratio (OR) and 95% confidence interval (CI) were also calculated. We investigated the independent relationship of all variables with a P-value < 0.2 in the univariate analysis using adjusted logistic regression method and provided crude and adjusted OR with their CI with a significance level of 0.05. Using the wealth index, we also applied the concentration index (C) and concentration curve (CC) to understand the socioeconomic-related inequality in the study population. In order to create CC, subjects are categorized based on their socioeconomic standing, and then the cumulative percentage of people is plotted against the cumulative percentage of unmet outpatient healthcare needs. CC above (below) the line of equality shows that health variable is concentrated among poor (rich) population. C values range from + 1 to − 1. Positive (negative) value shows that the health variable is concentrated among rich (poor) individuals, and C equaling zero means there is no inequality [18]. Data were analyzed using STATA software version 16.0 (Stata Corp, College Station, TX, USA.

## Results

### Descriptive results

In this study, 37.5% of subjects (505 out of 1346) perceived the need for outpatient health care services during the last 30 days, and their data were analyzed. Overall, out of 505 participants, 62.2% were female and only 12.7% of them were over 60 years old. In general, 8.5% of the participants were illiterate, and 61.0% were employed. 89.1% of the participants had basic health insurance coverage, and the economic status of 20.6% of the participants was classified as the poorest. Of this number, 31.7% (160 out of 505) had not received any formal outpatient health care services for their perceived needs. The characteristics of people who reported met and unmet needs for outpatient health care services are presented in Table 1. The highest perceived need is related to visiting a specialist doctor (21.4%) and using dental services (11.5%). Also, the highest met need was related to general doctor (70.7%) and specialist doctor visits (51.3%) and the highest unmet need was related to dental (80.6%) and rehabilitation services (78.8%).

**Table 1:** Characteristics of the study population reporting subjective met and unmet needs for outpatient healthcare services

Variables	Met 345 (68.3)	Unmet 160 (31.7)	*P*-value*
**Sex**			
Male	224 (71.3)	90 (28.7)	0.061
Female	121 (63.4)	70 (36.6)	
**Age**			
Under 30	102 (76.1)	32 (23.9)	0.027
30–59	206 (67.1)	101 (32.9)	
60 and above	37 (57.8)	27 (42.2)	
**Education**			
Illiterate	31 (72.1)	12 (27.9)	0.364
Primary	181 (71.3)	73 (28.7)	
Secondary	80 (64.5)	44 (35.5)	
Diploma and higher	53 (63.1)	31 (36.9)	
**Employment status**			
Employed	227 (73.7)	81 (26.3)	0.001
Unemployed	118 (59.9)	79 (40.1)	
**Basic health insurance**			
Yes	315 (70.0)	135 (30.0)	0.020
No	30 (54.5)	25 (45.5)	
**Complementary health insurance**			
Yes	24 (82.8)	5 (17.2)	0.085
No	321 (67.4)	155 (32.6)	
**Economic Status**			
The poorest	51 (49.0)	53 (51.0)	< 0.001
Poor	63 (63.0)	37 (37.0)	
Middle	64 (63.4)	37 (36.6)	
Rich	84 (84.0)	16 (16.0)	
The richest	83 (83.0)	17 (17.0)	

*P-value: Chi-square test

**Fig. 1:**
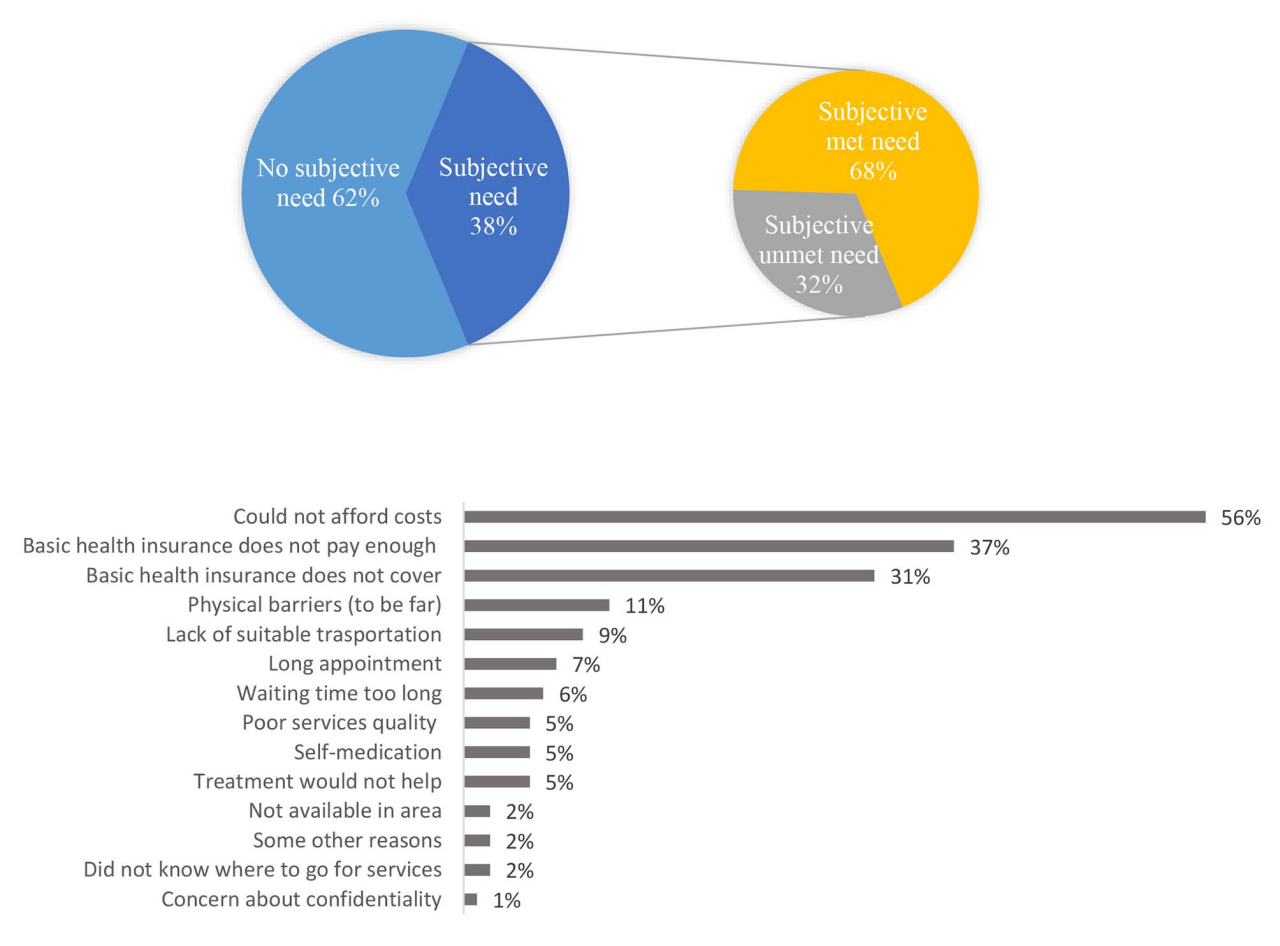
Reasons for subjective unmet need for outpatient healthcare services (Note: Every participant could choose more than one option)

The reasons for unmet outpatient healthcare needs are shown in Fig. 1. Lack of financial ability with 56%, insufficient payment of basic health insurances with 37%, lack of coverage of health services by basic health insurances with 31%, and lack of physical access with 11% were the main reasons for unmet outpatient healthcare needs (Fig. 1).

### Socioeconomic determinants of unmet outpatient healthcare needs

The results of the adjusted multiple logistic regression model are shown in Table 2. The variables of age group, employment status, and household economic status were significantly associated with odds of experiencing unmet outpatient healthcare needs. Being elderly with about 2.3 times (OR: 2.37, 95% CI: 1.19–4.75), not having a job with about 1.7 times (OR: 1.70, 95% CI: 1.13–2.57) and having a low economic status with about 4 times (OR: 4.46, 95% CI: 2.39–9.70) increased the odds of experiencing unmet outpatient healthcare needs (Table 2).

**Table 2:** Univariate and multivariate logistic regression models for subjective unmet need for outpatient healthcare services

Variables	Crude		Adjusted	
	OR (95% CI)	*P*-value	OR (95% CI)	*P*-value
**Sex**				
Male	1		1	
Female	1.43 (0.98–2.11)	0.062	1.44 (0.95–2.17)	0.080
**Age**				
Under 30	1		1	
30–59	1.56 (0.98–2.48)	0.059	1.88 (1.14–3.11)	0.013
60 and above	2.32 (1.23–4.39)	0.009	2.37 (1.19–4.75)	0.014
**Education**				
Illiterate	1		-	
Primary	1.04 (0.51–2.13)	0.911	-	
Secondary	1.42 (0.66–3.04)	0.366	-	
Diploma and higher	1.51 (0.67–3.36)	0.312	-	
**Employment status**				
Employed	1.87 (1.28–2.74)	0.001	1.70 (1.13–2.57)	0.011
Unemployed	1		1	
**Basic health insurance**				
Yes	1.44 (1.10–3.03)	0.022	1.80 (0.96–3.34)	0.063
No	1		1	
**Complementary health insurance**				
Yes	2.31 (0.86–6.19)	0.094	-	
No	1		-	
**Economic Status**				
The poorest	4.07 (2.05–8.07)	< 0.001	4.46 (2.39–9.70)	< 0.001
Poor	2.86 (1.485.55)	0.002	3.71 (1.85–7.41)	< 0.001
Middle	2.82 (1.45–5.46)	0.002	3.29 (1.67–6.49)	0.001
Rich	0.92 (0.44–1.96)	0.842	1.00 (0.46–2.14)	0.998
The richest	1		1	

**Fig. 2:**
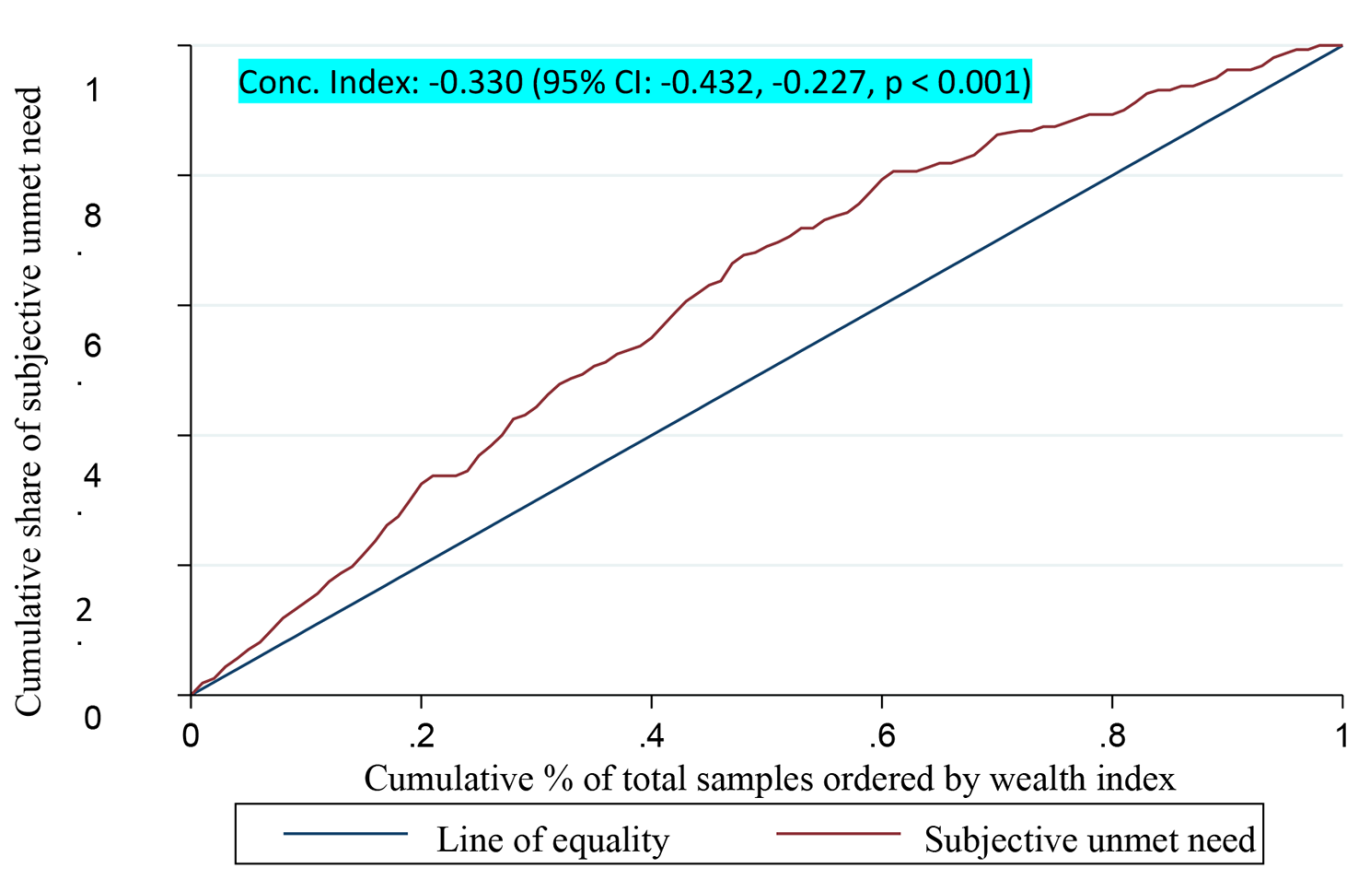
Concentration curve for subjective unmet need for outpatient healthcare services

The value of concentration index showed that unmet outpatient healthcare needs is significantly concentrated among people with lower economic status (C= -0.330, 95% CI: -0.432 to -0.227). As can be seen in Fig. 2, the concentration curve is placed above the line of equality; showing a more concentration of unmet outpatient healthcare needs among people with a more deprived economic status (Fig. 2).

## Discussion

The growing trend of unmet needs in informal settlements is a serious humanitarian crisis that authorities and policy makers of different countries are trying to overcome [2]One of the challenges of residents of informal settlements is lack of adequate access to health care services [7]. Understanding the unmet healthcare needs of the residents of informal settlements, identifying its causes and creating strategies to increase the access of these residents to needed healthcare services will help us have better cities to live in.

Based on the findings of this study, a significant percentage (about one third) of the need for outpatient health care services in informal settlements remains unmet. There is no similar study in Iran conducted on residents of these settlements for comparison, but this percentage is higher compared to the percentage reported in other studies conducted on general population of Iran. For instance, based on a national study among general population of Iran in 2016, the unmet outpatient healthcare needs was reported 17% [15]. In other studies conducted in developing countries, the percentage of unmet healthcare needs has been reported to be high and more than the general population [6, 7, 19]. The unmet need for women’s healthcare services in informal settlements in Kenya was reported to be more than 37% [20].

In this study, financial and physical barriers were the most important barriers to using outpatient healthcare services. This finding is consistent with the findings of other similar studies conducted in Iran [17]. In a national study conducted in 2016, the most important obstacle to using outpatient healthcare services was lack of financial access and provision of services [15]. According to our study, the highest percentage of unmet outpatient healthcare needs was related to dental and rehabilitation services. Poverty is one of the serious problems of residents of informal settlements, and this problem limits the household’s ability to pay for health expenses.

In addition, in Iran, dental and rehabilitation services are not covered by service package of basic health insurances [18]. This issue can lead to an increase in both unmet outpatient healthcare needs and out-of-pocket costs for these services. This finding indicates that having health insurance does not necessarily guarantee the use of services. About 90% of people participated in this study had basic health insurance. Also, weak service and cost coverage are other weaknesses of basic health insurance system in Iran [21].

In our study, the remoteness of the centers providing health services and the lack of a suitable public transportation system were other important barriers to use healthcare services. One of the problems of informal settlements is lack or shortage of health service providers in these areas. In developing countries, centers providing health services are not evenly distributed at the level of cities and are often concentrated in city centers [22, 23]. Due to illegal violations, informal settlements do not have access to basic health services, public transportation, roads, passages and electricity [6, 13]. Addressing the unmet healthcare needs of these settlements requires inter-sectoral cooperation between different actors in both public and private sectors.

According to the field visit by the research team of this study, the per capita index of health service providing centers such as comprehensive health centers, clinics, and pharmacies in informal settlements of Sanandaj city was very low compared to other parts of the city. So, in these settlements, the presence of government centers was very limited and the presence of the private sector was very weak. In Iran, most of the burden of outpatient healthcare services is on shoulders of private sector, and according to statistics, more than 70% of outpatient healthcare services are provided by this sector [17]. Therefore, it is suggested to have a fair distribution of health service centers in Sanandaj city with an emphasis on poor and deprived areas and neighborhoods and strengthen the public transportation system to increase the access of those living in informal settlements to city center.

As indicated by the findings of this study, the variables of being elderly, not having a job, and having a low economic status increased the odds of encountering unmet outpatient healthcare needs. In other similar studies in Iran, these variables have been reported as factors affecting the access and use of health services [15, 17]. Older individuals experience more health issues and have higher healthcare needs, which can increase the likelihood of encountering unmet outpatient healthcare needs. Moreover, age-related factors such as mobility limitations or cognitive decline can create barriers to accessing healthcare services [24]. Also it should be noticed that an unfriendly urban structure in urban informal settlements may contribute to an increase in unmet healthcare needs, including medical care requirements [24]. Not having a job or being unemployed removes a primary source of income for many people. Without a steady paycheck, it becomes difficult to afford copays, deductibles, prescriptions, and other out-of-pocket healthcare costs [25]. Unemployment, along with job loss, is associated with a decrease in preventative care-seeking behavior [26]. In addition, unemployment may endanger existing health insurance benefits obtained through an employer [25]. Our results showed that unmet outpatient healthcare needs were not equally distributed among different economic classes and it was mostly concentrated among poor groups of the society. Living in informal settlements disproportionately affects certain groups more. Unmet outpatient healthcare needs increase as the economic situation of informal settlements worsens, and poorer people are at higher risk in this regard [18]. Having a low economic status often associates with limited access to healthcare services. Financial limitations can lead to unmet needs for outpatient care, including difficulties in affording medications, transportation to healthcare facilities, or even co-pays for doctor visits which is associated with worse health outcomes and even premature death [24, 27, 28]. Considering the limited resources of the health sector, it is suggested to focus reform policies on poorer households. It is also necessary to design policies with the participation of all actors in order to target the root causes of poverty in these settlements and put them on the agenda of public policy makers.

The current study had some limitations. First, the data of this study was collected on the basis of self-report. It may be associated with recall bias, although the research team tried to reduce it by reducing the recall period to the last 30 days. Second, this study was conducted in one city; its findings may not be generalizable to the entire province and the entire country.

## Conclusion

Examining unmet healthcare needs can assist policymakers in evaluating access to healthcare services. There is a significant prevalence of unmet outpatient healthcare needs in informal settlements of Sanandaj city. A substantial portion of the residents in these settlements lacks access to necessary healthcare services. Disregarding the needs of individuals in these settlements, which constitute a large population of Iran, makes it difficult to achieve universal health coverage and other SDG3 goals. Financial barriers and inefficiencies in health insurance systems were identified as primary obstacles to accessing needed outpatient healthcare services in this study. Addressing these identified barriers requires the participation of all stakeholders, including the government, the nation, and civil society. To improve access to outpatient healthcare services for residents in these areas, policy recommendations include considering health subsidies, exemptions for low-income households from healthcare costs, establishing mobile clinics in these areas, and ensuring equitable distribution of healthcare facilities within the city. Additionally, creating suitable public transportation to facilitate access to urban centers is suggested. By implementing these policy suggestions, the aim is to enhance access to necessary outpatient healthcare services for the residents of these areas and work towards achieving equitable healthcare provision.

## Data Availability

The datasets used and/or analyzed during the current study are available from the corresponding author on reasonable request.
